# Inadvertent Implantation of Pacemaker Lead in the Left Ventricle: Kill Two Birds with One Stone

**Published:** 2014-04-01

**Authors:** Ugur Onsel Turk, Esref Tuncer, Emin Alioglu, Istemihan Tengiz, Ertugrul Ercan

**Affiliations:** 1 Department of Cardiology, Central Hospital, Izmir, Turkey; 2 Department of Cardiology, Izmir University, Izmir, Turkey

**Keywords:** Pacemaker, Embolization, Anticoagulant

## Abstract

We report an asymptomatic patient in whom the intravenous pacemaker (PM) lead was inadvertently implanted in LV through the perforated interventricular septum. He had no embolic events during the last 9 years after the implantation. Possible explanation of the uncomplicated follow-up period is that the patient had been taking warfarin because of mechanical mitral valve prosthesis.

## 1. Introduction

Inadvertent ventricular lead implantation into the Left Ventricle (LV) is a known complication of permanent pacing. This may be caused by perforation of the interventricular septum and migration of the lead into the LV, which carries a high risk of systemic embolization. Here, were port an asymptomatic patient in who man intravenous Pacemaker (PM) lead was in advertently implanted in the LV through the perforated interventricular septum. He had no embolic events within 9 years after the implantation. Possible explanation of the uncomplicated follow-up period is that the patient had been taking warfarin because of mechanical mitral valve prosthesis.

## 2. Case Report

**Figure 1.**
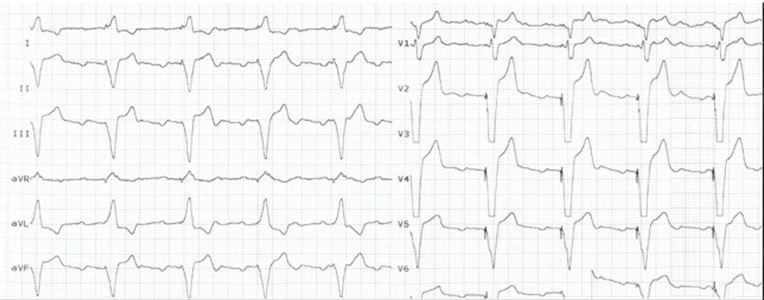
Twelve-Lead ECG Which Demonstrated Pacemaker Rhythm and the LBBB Pattern of QRS Complexes

**Figure 2.**
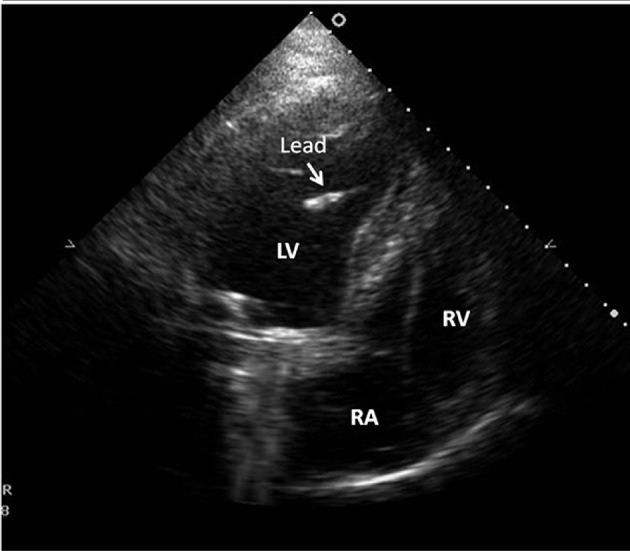
Fluoroscopic View of Pacing Lead LV, Left Ventricle; RV, Right Ventricle; RA, Right Atrium The white arrow indicates pacemaker lead tip in the LV.

**Figure 3.**
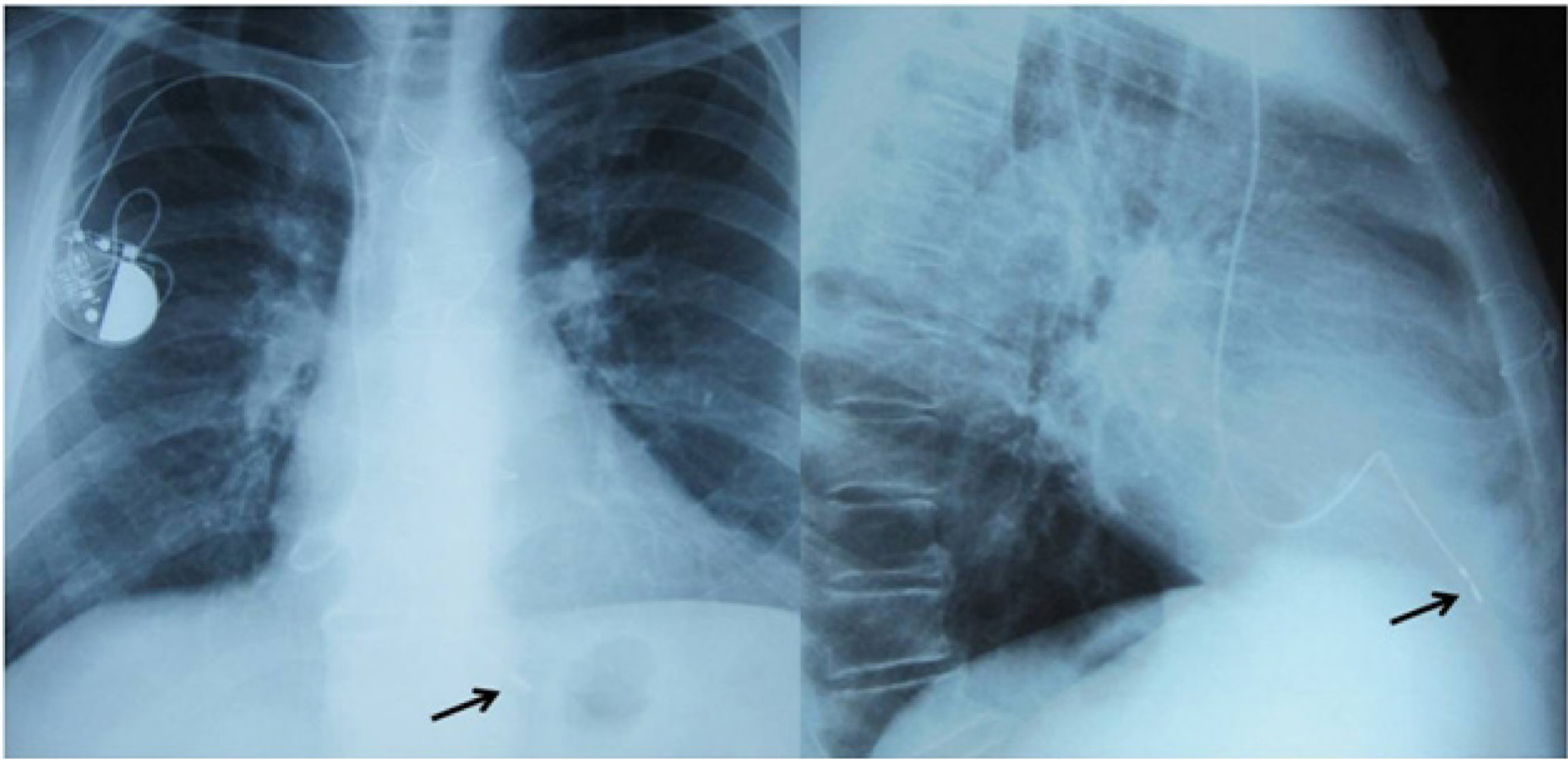
Postero-Anterior and Lateral Chest X-ray Black arrows indicate pacemaker lead tip in the LV.

A 73-year-old asymptomatic patient admitted to our center for routine PM follow-up. He had a history of mechanical mitral valve implantation 12 years ago and VVIR PM implantation 9 years ago. At presentation, his physical examination was unremarkable. Pertinent laboratory data, including complete blood count, renal function test, and liver function test, were also within the normal limits. Additionally, prothrombin times, including the last six months, were in the therapeutic window. ECG demonstrated PM rhythm with QRS complexes in Left Bundle Branch Block (LBBB) morphology. Additionally, detailed analysis of V_2_ derivation exhibited small “r” waves (Figure 1). Moreover, Transthoracic Echocardiogram (TTE) analysis showed a normal function in mechanical mitral valve prosthesis butan abnormal route of the PM lead. The lead had passed through the perforated apical interventricular septum from the right ventricle to the the Left ventricle (Figure 2). Anteroposterior and lateral chest X-rays suggested that the position of the distal portion of the lead was in the apical segment of the LV (Figure 3). Pacemaker capture and sensing thresholds (0.5 V and 21 mV, respectively) and lead impedance (662 ohm) were within the normal limits. We decided to remove the malpositioned lead in the LV because of the patient’s age and non-embologenic history and that he was receiving anti coagulant therapy.

## 3. Discussion

Erroneous ventricular pacing lead in the LV is a rare complication of PM implantation. It carries a high risk of systemic embolization, mainly in the form of recurrent transient ischemic attack or stroke (1). Although early diagnosis of the complication can be made using ECG, X-ray, or echocardiography, sometimes it may be overlooked. In these cases, the usual presentation of the complication is thromboembolic events. In general, the left heart lead placement diagnosed late after the implantation can be treated by either anticoagulant therapy or lead removal. Anticoagulant therapy with warfarin seems to be effective in primary and secondary prevention of thromboembolic events (2).

In the present study, the patient was very lucky because he was taking warfarin for mechanical mitral valve prosthesis. We assume that probably warfarin therapy had prevented the patient from systemic embolic complications. Another striking face of the case was QRS morphology. LV pacing produces a positive QRS deflection on leads V_1_ and V_2_, butanegative deflection on lead I. On the other hand, normal RV pacing shows a negative deflection on V_1_ and V_2_ and a positive deflection on lead I (3). In the first glance, the patient’s ECG was characterized with RV pacing. Nevertheless, comprehensive evaluation of QRS pattern in V_2_ showed a small initial positive deflection in this lead. This unusual morphology suggested that the initial electrical activation of the ventricle site was LV endocardium. However, this is not a rule and detailed analysis of ECG may give more valuable clues. Overall, when suspicion exists regarding the lead position, TTE should be used to confirm the location of the leads.
